# A BODIPY-Based Fluorogenic Probe for Specific Imaging of Lipid Droplets

**DOI:** 10.3390/ma13030677

**Published:** 2020-02-03

**Authors:** Guanglei Li, Jianye Li, Yu Otsuka, Shuai Zhang, Masashi Takahashi, Koji Yamada

**Affiliations:** 1 Division of Materials Science, Graduate School of Environmental Science, Hokkaido University, Sapporo 0860-0810, Japan; y_outsuka214@eis.hokudai.ac.jp (Y.O.); zs929783910@eis.hokudai.ac.jp (S.Z.); 2 Laboratory of Animal Genetics and Reproduction, Graduate School of Agriculture, Hokkaido University, Sapporo 060-8589, Japan; lijianye218@163.com; 3 Laboratory of Animal Genetics and Reproduction, Research Faculty of Agriculture, Hokkaido University, Sapporo 060-8589, Japan; mmasashi@anim.agr.hokudai.ac.jp; 4 Graduate School of Global Food Resources/Global Station for Food, Land and Water Resources, Global Institution for Collaborative Research and Education, Hokkaido University, Sapporo 060-8589, Japan

**Keywords:** lipid droplet, BODIPY, fluorogenic probe

## Abstract

We developed an easily accessible boron-dipyrromethene (BODIPY)-based fluorogenic probe, which we named LD-TB. This probe emits bright fluorescence in oil; when compared with aqueous solution, a significant enhancement of fluorescence brightness is observed. Cellular experiments confirmed that the probe stains the lipid droplets (LDs) specifically in both live and fixed cells, providing background-free images. Compared with Nile Red dye, a commonly used LD marker, LD-TB showed superior photostability. The sharp absorption and emission bands enable its multicolor imaging with blue and green probes. Importantly, the probe has proved to have low toxicity and is compatible with cell fixation. Our research provides a promising new fluorogenic probe for specific imaging of LDs.

## 1. Introduction

Lipid droplets (LDs) are intracellular organelles that store neutral lipids within cells, including triglycerides and cholesterol ester [1]. The LDs are ubiquitous organelles and are found in most eukaryotic cells and in some prokaryotes [2]. Long perceived as mere inert reservoirs of neutral lipids, LDs are now considered as fully entitled organelles that represent a new frontier in cell biology [3,4]. In recent years, a considerable number of research studies have shown that LDs are of great importance in many physiological processes, such as membrane trafficking [5], protein degradation [6], and inter-organelle communication [7]. It has become increasingly evident that LDs play a very important role in health and disease and that abnormalities in LDs could lead to many serious diseases [8,9]. For instance, LDs are critical in the development of lipoatrophy, a condition that leads to lipoatrophic diabetes in mice and humans [10]; it is crucial, therefore, to identify and monitor cellular LDs effectively.

Fluorescence techniques are indispensable tools for biological applications, given their considerable merits when compared with other measurement methods, especially for sensing and visualizing of analytes inside living systems [11]. Fluorescent dyes correspond to conversion of invisible biological signals to detectable fluorescent signals. It is noticeable that turn-on dyes, which turn on their emission light only after binding to targets, provide high signal-to-noise imaging [12,13,14,15].

Nile Red and BODIPY 493/503 are the most commonly used LD markers. However, in tests, both dyes showed some drawbacks. Nile Red is known to non-specifically stain other organelles and to display broad absorption and emission bands [16], making it unsuitable for multicolor imaging. BODIPY 493/503 is reported to have limited photostability [17], which compromises its application for spatial and temporal resolution. Although, new fluorescent probes for the visualization of LDs have been developed, including aggregation-induced emission (AIE) fluorophores [18,19,20,21], merocyanines [22], fluoranthenes [23], and solvatochromic dyes [24,25,26], turn-on LD-specific dyes are very limited, especially for emitting in red to the NIR region [22,27,28,29]. Most recently, a family of turn-on LD markers were developed on the basis of merocyanine, which showed high signal-to-noise imaging of LDs in cells and tissues; however, they were used for a prolonged incubation time (3 h) probably due to their poor membrane-penetration properties [22]. It is imperative, therefore, to develop an efficient molecular probe specifically for LDs.

Herein, we present an easily accessible BODIPY-based fluorogenic probe for the specific staining of LDs. The probe, named LD-TB, emits bright red fluorescence due to its high fluorescence quantum yield and high molar absorption coefficient. Since the fluorescence decreased dramatically in aqueous mixture, LD-TB showed high signal-to-noise imaging. Its high photostability and low toxicity to cells allow its wide application in the biological field. In addition, because of the sharp absorption and emission bands, multicolor imaging of the red emission LD-TB with blue and green fluorescence dyes was easily achieved by using commercially available filter sets.

## 2. Materials and Methods

### 2.1. Synthesis

Commercially available chemicals for synthesis were purchased from Wako (Richmond, VA, USA), TCI (Tokyo, Japan) or Sigma-Aldrich (St. Louis, MO, USA) and used as received. NMR spectra were recorded on a JEOL 400 spectrometer (Tokyo, Japan) at room temperature using tetramethylsilane (TMS) as internal standard (δ = 0 ppm). Mass spectrum was obtained on a Thermo Scientific Exactive (Waltham, MA, USA) under electrospray ionization (ESI) condition. Melting point was measured with a Yamato MP-21 apparatus. Detail procedures of synthesis and characterization are described in the Supplementary Information.

### 2.2. Spectroscopy

The organic solvents used for spectroscopy measurement were spectra grade, and the water was Milli-Q water (Millipore, Burlington, MA, USA). Sunflower oil, stabilized with about 0.02% of d-δ-tocopherol, was obtained from Wako. The UV-Vis absorption spectra were performed on a JASCO V-770 spectrophotometer (JASCO Group, Tokyo, Japan), and the fluorescence spectra and quantum yields were obtained using a Hamamatsu Photonics Quantaurus-QY Absolute PL quantum yield spectrometer C11347 (Hamamatsu Photonics, Yokohama, Japan). Dynamic light scattering (DLS) measurements were performed on a Zetasizer Nano ZS analyzer (Malvern Instruments Ltd., Malvern, UK).

### 2.3. Cell Imaging

Bovine cumulus cells were cultured in culture medium (DMEM with 5% FBS) at 38.5 °C in a humidified atmosphere of 95% air and 5% CO_2_. Working solution of the dyes were diluted to certain concentration with culture medium or PBS, and used to stain the cells for specified time. The cells were imaged under a Leica DMi8 fluorescence microscope (Leica Camera AG, Wetzlar, Germany)) using proper excitation and emission filter cubes for each dye: for Hoechst 33342, DAPI filter set (excitation filter 350/50 nm, dichroic mirror 400 nm, emission filter 460/50 nm), for LipiDye, DAPI LP filter set (excitation filter 360/40 nm, dichroic mirror 400 nm, emission filter 425 nm longpass), for MitoTracker Green FM, L5 filter set (excitation filter 480/40 nm, dichroic mirror 505 nm, emission filter 527/30 nm), for Nile Red and LD-TB, TX2 filter set (excitation filter 560/40 nm, dichroic mirror 595 nm, emission filter 645/75 nm). The images were recorded with Leica Application Suite and processed using ImageJ 1.52a software (NIH, Bethesda, MD, USA).

### 2.4. Cell Viability

The cell viability was measured with the Cell Counting kit-8 (CCK-8) assay, which utilizes WST-8 to measure the activity of dehydrogenase in cells. Cells were seeded in a 96-well plate at a density of 5 × 10^3^ cells/well in culture medium at 38.5 °C in a humidified atmosphere of 95% air and 5% CO_2_. After 1 day, the medium culture was replaced with 100 µL fresh medium containing 0, 0.05, 0.5, 2, 5, and 10 µM of LD-TB and incubated at 38.5 °C. The final volume fraction of DMSO was 0.5%, and empty well filled with the same volume of 100 µL fresh medium were set for a blank control. After for 48 h incubation, 10 µL of CCK-8 solution was added to each well and further incubation at 38.5 °C for 1 h. The absorbance was measured at 450 nm using a microplate reader. For each concentration, the percentage of the cell viability was calculated in the reference to the control group in the absence of LD-TB.

### 2.5. Photostability

The photostability of the dyes in the cells were evaluated on fixed bovine cumulus cells. After reaching 70–80% of confluency, the cells were incubated with LD-TB (0.05 µM) at 38.5 °C for 30 min or Nile red (0.5 µM) at room temperature for 1 h, and then fixed with 4% paraformaldehyde (PFA) for 15 min at room temperature. The stained cells were irradiated and imaged by using TX2 filter cube set, and repeated scans were taken continuously. The regions of interest (ROIs) of each scan were defined, and the pixel intensity of each scan was referred to that of each first scan.

## 3. Results

### 3.1. Synthesis and Photophysical Studies of LD-TB

**Scheme 1:**
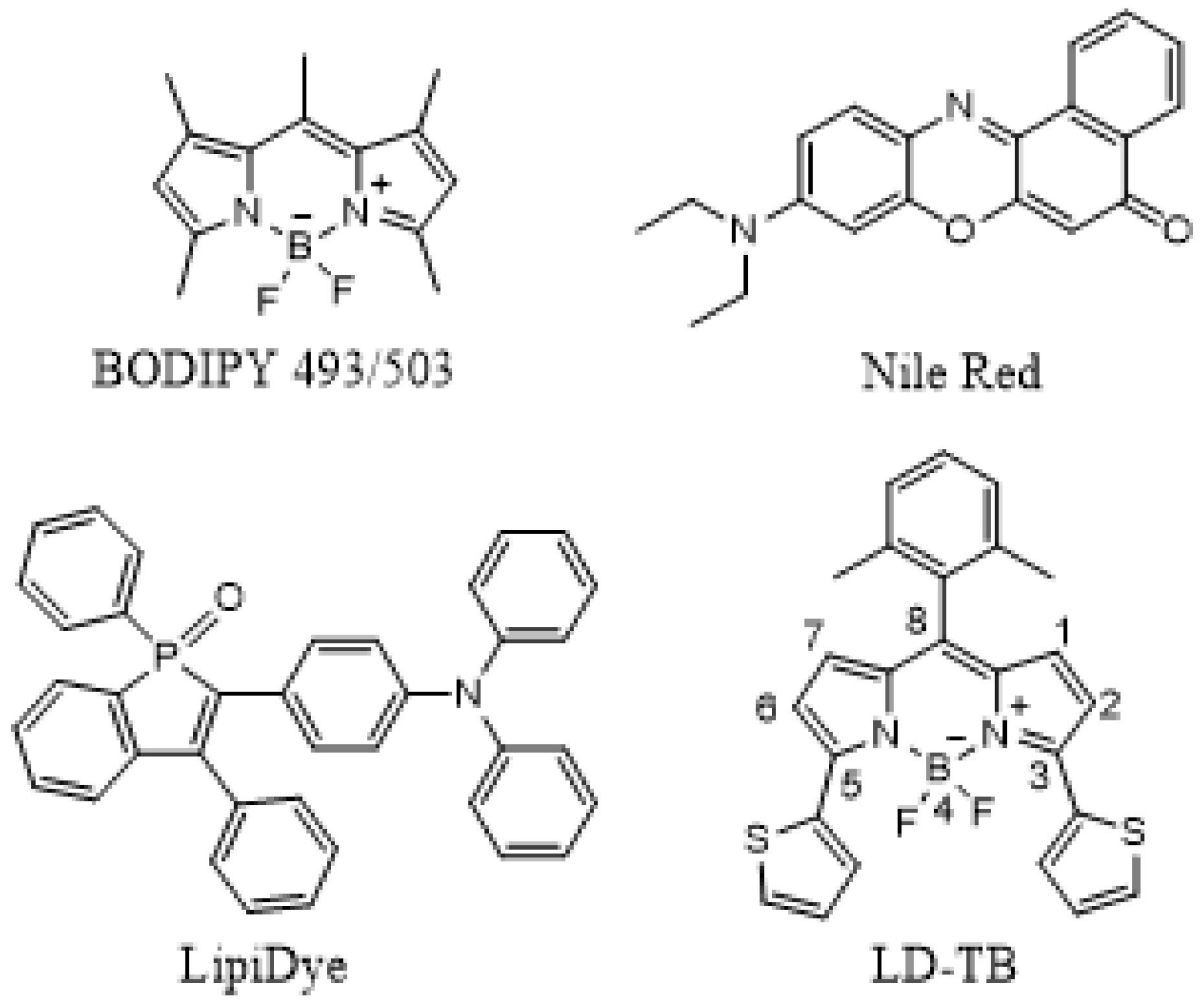
Structures of LD probes: BODIPY 493/503, Nile Red, LipiDye, LD-TB.

**Figure 1:**
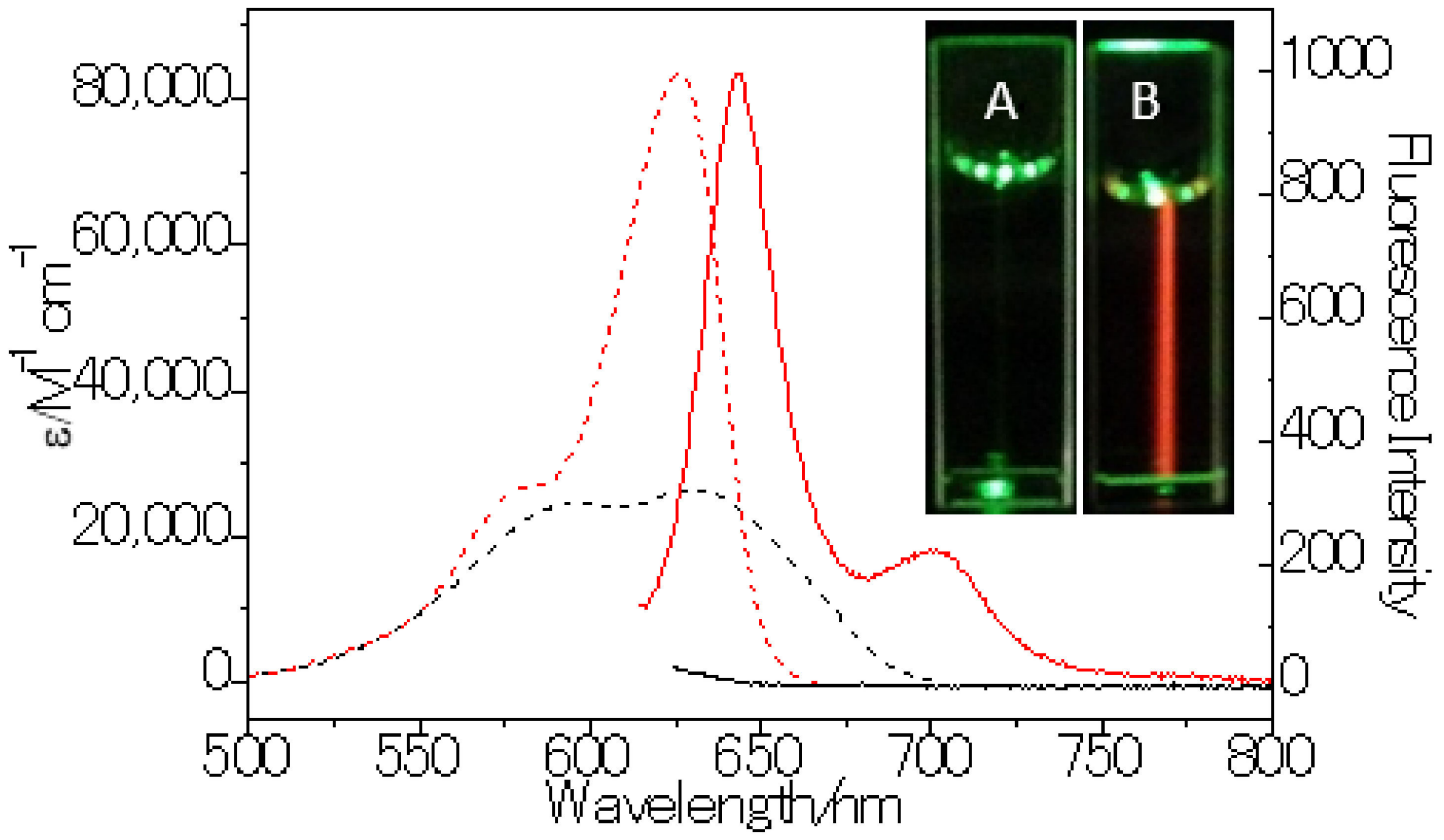
Normalized absorption (dash line) and emission spectra (solid line) of LD-TB (1 µM) in 0.5% DMSO/H_2_O (black) and sunflower oil (red). Inset: Images of LD-TB in 0.5% DMSO/H_2_O (**A**) and sunflower oil (**B**), excitation light: 530 ± 10 nm, images were captured with no detection filter.

We found that one of our previously synthesized BODIPY probes showed good performance as a red turn-on LD marker. The easily accessible fluorogenic probe LD-TB (8-(2,6-dimethylphenyl)-3,5-bis(2-thienyl)-4,4-difluoro-4-bora-3a,4a-diaza-s-indacene), as shown in Scheme 1, was synthesized via a one-step Suzuki–Miyaura cross-coupling reaction [30]. BODIPY is known for its high hydrophobicity and outstanding photophysical properties, and the 2,6-dimethylphenyl group installed at the 8-position was aimed to achieve high fluorescence quantum yield by restricting the intermolecular rotation [31]. Thiophene groups modified to the 3,5-position are known to prolong the wavelengths toward long region [32] meanwhile maintain a small molecular size. The structure of LD-TB was confirmed by ^1^H NMR, ^13^C NMR, and mass spectrometry (Figures S1–S3). LD-TB has been reported to emit bright fluorescence in the red wavelength region in dichloromethane [30]. To further explore its photophysical properties, we investigated its optical properties in five kinds of organic solvent (Figure S4) as well as in 0.5% DMSO/H_2_O solution and sunflower oil (Figure 1); the data are summarized in Table 1.

LD-TB showed typical photophisical properties in five kinds of organic solvent, such as good fluorescence quantum yield, high molar absorption coefficient, and moderate Stokes-shift. It also displayed similar absorption and emission spectra bandswith only a slight redshift (~10 nm) from cyclohexane to DMSO. The emission maxima of LD-TB are not sensitive to the change in solvent polarity; this is probably because both the lowest unoccupied molecular orbital (LUMO) and the highest occupied molecular orbital (HOMO) are centered largely around the BODIPY core [33]. It is worth mentioning that the absorption and emission bands with the full width at half maximum (FWHM) is about 27 nm. The relative sharp absorption and emission bands would be beneficial in preventing cross-talk with other fluorescent dyes.

Because of its high lipophilicity, LD-TB cannot be dissolved in H_2_O but suspends well in 0.5% DMSO/H_2_O (v/v). In this condition, it demonstrated a broader absorption band with a significantly decreased molar absorption coefficient than in the organic solvents. The broader absorption band suggested that LD-TB formed soluble aggregated particles in the DMSO/H_2_O mixture, which is further confirmed by the dynamic light scattering (DLS) measurement (Figure S5). Consequently, a dramatic decrease in fluorescence quantum yield was observed (<0.01). The dye was non-emissive in the DMSO/H_2_O mixture, probably because the hydrophobic aromatic LD-TB dye is liable to aggregate in the high-portion aqueous solution and triggered an aggregation-caused quenching (ACQ) effect [29,34]. Sunflower oil (mainly of unsaturated long-chain triglycerides) was used to simulate the condition in LDs. As expected, the dye displayed similar spectroscopic properties in the oil to those in the organic solvents. For example, high fluorescence quantum yield and brightness and sharp absorption and emission bands. Consequently, it achieved an impressive fluorescence enhancement (>270-fold) compared with the DMSO/H_2_O mixture solution.

### 3.2. LD-TB Stains Specifically Lipid Droplets in Cells

**Figure 2:**
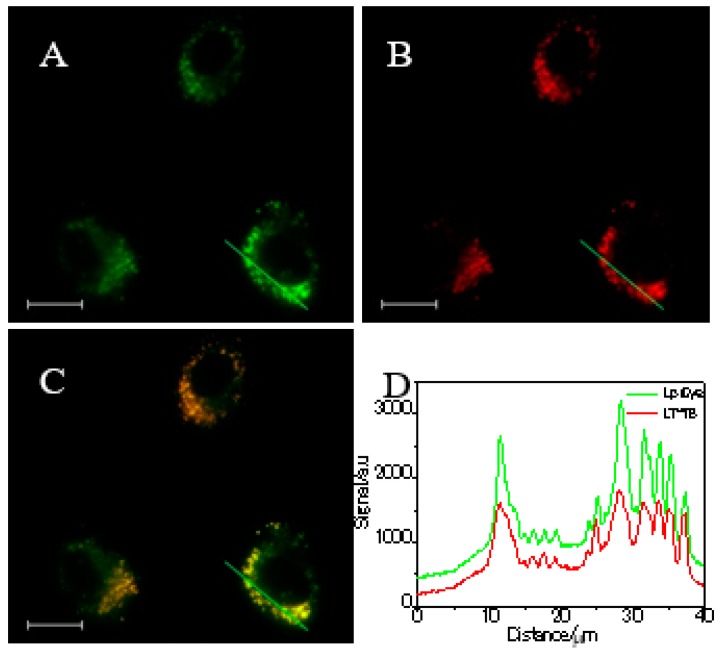
Fluorescence images of bovine cumulus cells stained with (**A**) LipiDye (3 µM) for 2 h and (**B**) LD-TB (0.05 µM) for 30 min. (**C**) Merged images of A and B. (**D**) Intensity profile of ROIs across bovine cumulus cells (the green line in (**A**–**C**)). Scale bar: 20 µm.

Given its interesting photophysical properties in oil and aqueous solutions, we explored the utility of LD-TB as a fluorescent probe for cell imaging. Bright fluorescence images were obtained after incubation bovine cumulus cells with 0.05 µM LD-TB for 30 min at 38.5 °C (Figure S6). On the basis of morphological grounds, the moving reticles were expected to be LDs. To verify our speculation, a co-localization experiment with LipiDye (a green marker for LDs) was performed and a good overlap image was obtained by merging the fluorescence images of the two dyes captured from two different channels (Figure 2, Figure S7). Moreover, the intensity profile of the linear region of interests (ROIs) for the two dyes across the cell varied in similar tendencies, which suggests a high overlap between the two dyes. Pearson’s correlation factor was used to quantify the staining region overlap of the two dyes. A high Pearson’s correlation coefficient of 0.89 was observed, confirming a good specificity of LD-TB to LDs. The high specificity of the LD-TB accumulated in LDs was due to the like–like interactions between the hydrophobic LD-TB dye and the hydrophobic LDs [19]; no fluorescence noise was generated from the background because the LD-TB is non-emissive in aqueous cytoplasm.

### 3.3. LD-TB is Not Cytotoxic to Live Cells

**Figure 3:**
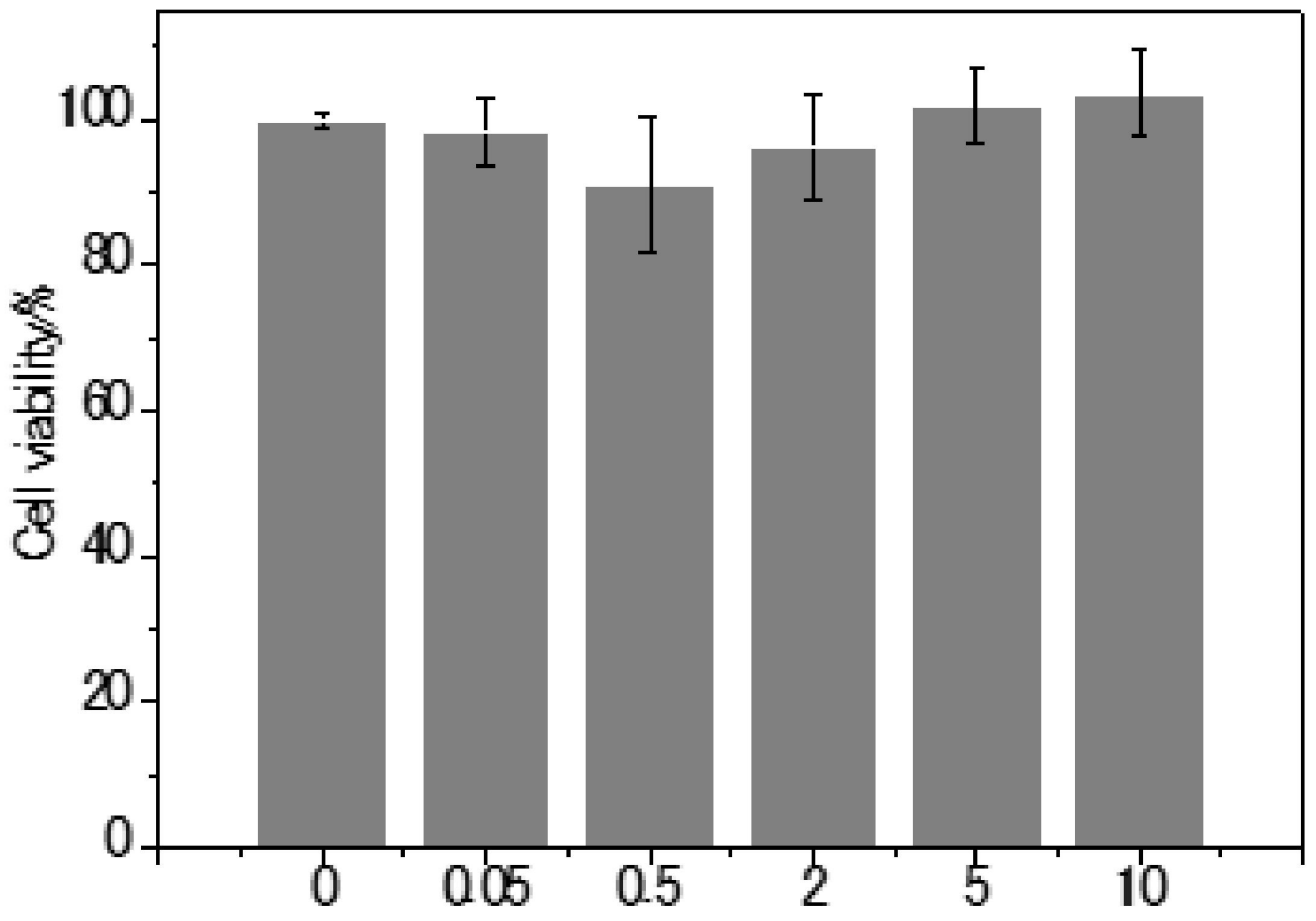
Cytotoxicity of LD-TB at various concentrations to living bovine cumulus cells.

To investigate the cell membrane penetration efficiency of LD-TB, the images of both live and fixed cells were taken at various incubation times (Figure 3). The fluorescence in LDs enhanced over incubation time, indicating a high penetration efficiency of LD-TB for live cells. The results showed that LD-TB is also applicable for fixed cells staining at room temperature, although at lower efficiency. We examined the cytotoxicity of LD-TB to bovine cumulus cells during a prolonged (48 h) incubation period, based on CCK-8 assay. As suggested in Figure S10, no significant decrease in cell viability was observed up to a high concentration of 10 µM (up to 200-fold the working concentration), suggesting that the dye is cytocompatible and has almost no cytotoxicity.

### 3.4. LD-TB Displays High Photostability

**Figure 4:**
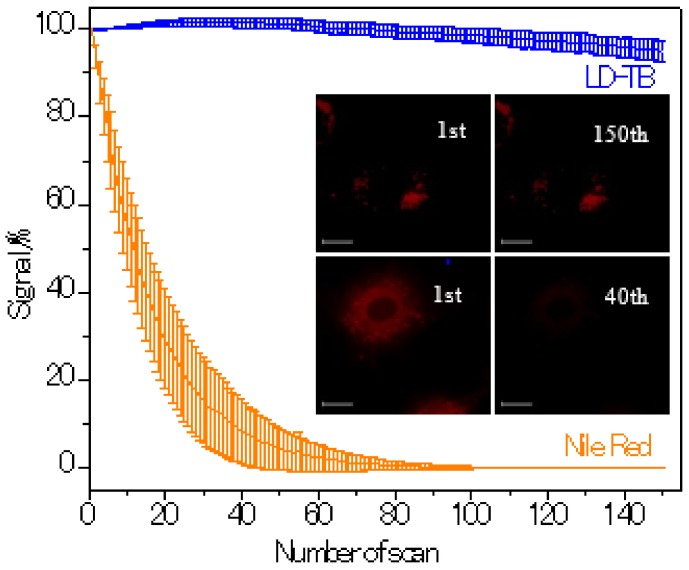
Fluorescence intensity change of LD-TB and Nile Red in bovine cumulus cells with a number of scans. Inset: Images of bovine cumulus cells stained with LD-TB (upper) and Nile Red (lower), which were fixed with paraformaldehyde (PFA), obtained upon continuous scanning under identical irradiation conditions. Concentration: 0.05 µM for LD-TB, 0.5 µM for Nile Red. Incubation time: 30 min for LD-TB, 60 min for Nile Red. Scale bar: 20 µm.

The photostability of LD-TB in bovine cumulus cells was then evaluated by using Nile Red, a commonly used red LD marker, for comparison. Since live cells are dynamic, to simplify data processing, stained cells were fixed with paraformaldehyde (PFA) before scanning (Figure S9). The intensity loss of the dyes was used to quantitatively represent the photostability. As depicted in Figure 4, after 150 scans, the fluorescence intensity loss of LD-TB was less than 5%, and no significant change was observed between the original and the 150th scanning images. More than half the fluorescence intensity of the Nile Red was lost in only 12 scans. The results suggest that LD-TB is resistant to photobleaching and is suitable for long-term monitoring of LDs.

### 3.5. Multicolor Imaging of LD-TB

**Figure 5:**
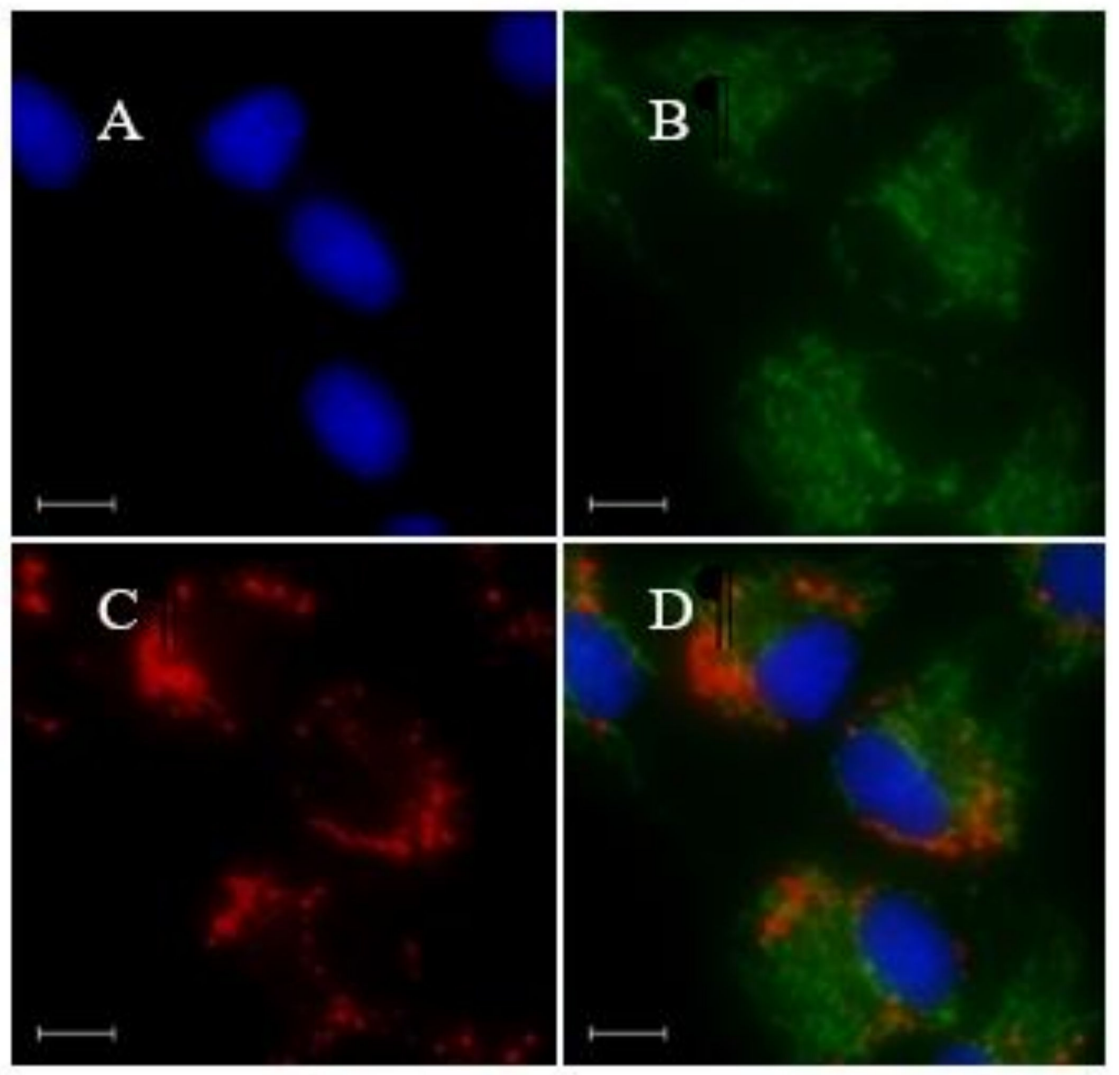
Fluorescence images of bovine cumulus cells stained with (**A**) Hoechst 33342 (1 µg/mL), (**B**) MitoTracker Green FM (0.2 µM), (**C**) LD-TB (0.05 µM), incubation for 30 min. (**D**) Merged images of (**A**–**C**). Scale bar: 10 µm.

A multicolor imaging study was conducted by co-staining LD-TB with Hoechst 33342 (a blue marker for nucleus) and MitoTracker Green FM (a green marker for mitochondria). As is shown in Figure 5, due to the sharp absorption and emission bands, combined with negligible solvatochromism of LD-TB, no cross-talk with the emissions from the other two dyes was observed. The experiments suggest that LD-TB as a red LD marker is suitable for multicolor imaging with blue and green fluorescent dyes, which could greatly extend its application in cell imaging.

## 4. Conclusions

In this study, we discovered a versatile BODIPY-based fluorogenic probe for LD-specific staining. The probe, named LD-TB, emits bright red fluorescence in oil and has an impressive emission enhancement compared with that in aqueous conditions. LD-TB selectively stained the LDs in both live and fixed cells and presented bright and background-free imaging, with no obvious cytotoxicity. Importantly, LD-TB had strong photostability in cells, and the multicolor imaging was easily approached because of its sharp absorption and emission bands. When considered together, these factors validate LD-TB as a practical LD marker, indicating its potential application for LD-related research. The outstanding performances of the small molecular BODIPY probe have encouraged us to do further modification of the BODIPY probe for bio-imaging.

## Figures and Tables

**Table 1:** Photophysical properties of LD-TB (1 µM) in various solvents.

Solvent	λ_abs_ /nm	ε /M^−1^ cm^−1^	FWHM_abs_ /nm	λ_em_ /nm	FWHM_em_ /nm	Φ	Stokes shift/nm	Brightness (ε × Φ)
Cyclohexane	621	83,000	50	638	23	0.84	17	69,720
Toluene	625	74,000	42	644	27	0.90	19	66,600
Ethyl acetate	617	73,000	59	638	27	0.85	21	62,050
Acetone	617	72,000	56	639	27	0.85	22	61,200
DMSO	628	69,000	44	650	29	0.84	22	57,960
0.5% DMSO/H_2_O	630	26,000	105	-	-	<0.01	-	<260
Sunflower oil	626	84,000	38	643	26	0.85	17	71,400

## Supplementary Materials

The following are available online at https://www.mdpi.com/1996-1944/13/3/677/s1, Figure S1: ^1^H NMR spectrum of LD-TB (400 MHz, CDCl_3_) at room temperature, Figure S2: ^13^C NMR spectrum of LD-TB (100 MHz, CDCl_3_) at room temperature, Figure S3: Mass spectrum of LD-TB, Figure S4: The normalized absorption spectra (left) and normalized emission spectra (right) of LD-TB (1 µM) in various organic solvents, Figure S5: DLS measurements of LD-TB in 0.5% DMSO/H_2_O at 0.05 µM, Figure S6: Fluorescence images of bovine cumulus cells stained with 0.05 µM LD-TB 30 min. Different false color (green, red, blue) are used to present the images captured at different times of 0, 1, 5 min (**A**–**C**). Merged images at different times, 0 and 1 min (**D**), 1 and 5 min (**E**), respectively. Bright field (**F**). Movement of the stained vesicles can be easily observed in the merged images. Scale bar 25 µm, Figure S7: Fluorescence images of bovine cumulus cells: (**A**,**B**) stained with 0.05 µM LD-TB 30 min, (**C**,**D**) stained with 3 µM LipiDye 2 h, (**E** and **F**) stained with both dyes, firstly 3 µM LipiDye 1.5 h and then co-stain with 0.05 µM LD-TB for further 30 min. No cross-talk was observed in the colocalization experiments. Scale bar: 20 µm, Figure S8: Images of live and fixed bovine cumulus cells stained with LD-TB (0.05 µM) for different period of time. For live cells, they were incubated at 38.5 °C. For fixed cell, they were fixed, washed and then incubated with LD-TB at room temperature. Conditions for images recording and post-processing were identical. Scale bar: 25 µm, Figure S9: Images of bovine cumulus cells incubated with LD-TB (0.05 µM) for 30 min of live cells (**A**–**C**), and the images of cells incubated with LD-TB (0.05 µM) for 30 min of live cells and then fixed with PFA (**D**–**F**). (**A**,**D**) Bright field images. (**B**,**E**) Fluorescence images. (**C**,**F**) Image J 3D surface plot analysis. Conditions for images recording and post-processing were identical. No fluorescence quenching caused by fixation process was observed. Scale bar: 25 µm.
