# Supplementary material for: A BODIPY-Based Fluorogenic Probe for Specific Imaging of Lipid Droplets
Source: Materials (Basel). 2020 Feb 3;13(3):677. doi: 10.3390/ma13030677 (PMC7040634; doi:10.3390/ma13030677)
Supplement: Supplementary file 1 [file materials-13-00677-s001.pdf]

materials

Supplementary

A BODIPY-Based Fluorogenic Probe for Specific Imaging of Lipid Droplets

Guanglei Li [1,*], Jianye Li [2], Yu Otsuka [1], Shuai Zhang [1], Masashi Takahashi [3,4] and Koji Yamada [1,*]

[1] Division of Materials Science, Graduate School of Environmental Science, Hokkaido University, Sapporo 0860-0810, Japan; y_outsuka214@eis.hokudai.ac.jp (Y.O.); zs929783910@eis.hokudai.ac.jp (S.Z.)
[2] Laboratory of Animal Genetics and Reproduction, Graduate School of Agriculture, Hokkaido University, Sapporo 060-8589, Japan; lijianye218@163.com
[3] Laboratory of Animal Genetics and Reproduction, Research Faculty of Agriculture, Hokkaido University, Sapporo 060-8589, Japan; mmasashi@anim.agr.hokudai.ac.jp
[4] Graduate School of Global Food Resources/Global Station for Food, Land and Water Resources, Global Institution for Collaborative Research and Education, Hokkaido University, Sapporo 060-8589, Japan
* Correspondence: ligliht@gmail.com (G.L.), yamada@ees.hokudai.ac.jp (K.Y.); Tel +81-011-706-2254 (K.Y.)

Received: 17 January 2020; Accepted: 1 February 2020; Published: 3 February 2020

Synthesis of 8-(2,6-dimethylphenyl)-3,5-bis(2-thienyl)-4,4-difluoro-4-bora-3a,4a-diaza-s-indacene (LD-TB)

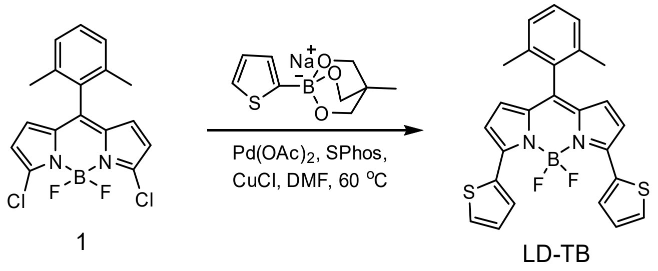

LD-TB was synthesized according to an earlier reported protocol[1].

3,5-dichloroBODIPY **1** (91 mg, 0.25mmol), (2-Thiophene)cyclic-triolborate Sodium Salt (175 mg, 0.75mmol), Palladium(II) acetate [$Pd(OAc)_2$] (6 mg, 0.027 mmol), 2-Dicyclohexylphosphino-2′,6′-dimethoxybiphenyl (SPhos) (12 mg, 0.029 mmol), and CuCl (0.10 mmol) were placed in a 10 ml two-necked round bottom flask. The flask was purged with N_2 gas three times, and then added *N,N*-dimethylformamide (DMF) 2.5 mL. After stirring at 60 °C for 20 h, the reaction mixture was cooled to room temperature, then extracted with ethyl acetate and washed with brine successively. The organic layer was dried over Na_2SO_4, evaporated and the crude sample was purified by silica gel column chromatography using gradient eluent with eluent pair toluene/hexane, to give LD-TB (48 mg, 42%) as a dark red solid.

Rf = 0.22 (toluene:hexane 1:2), M.p. 218–220 °C

^{1}H NMR (400 MHz, $CDCl_3$): δ (ppm) = 8.23 (dd, *J* = 3.8 Hz, *J* = 0.8 Hz, 2H, thio-H), 7.48 (dd, *J* = 5.4 Hz, *J* = 0.8 Hz, 2H, thio-H), 7.30–7.25 (m, 1H, Ph-H), 7.2–7.19 (m, 2H, thio-H), 7.14 (d, *J* = 7.6 Hz, 2H, Ph-H), 6.75 (d, *J* = 4.3 Hz, 2H, Pyr-H), 6.53 (d, *J* = 4.3 Hz, 2H, Pyr-H), 2.19 (s, 6H, CH_3).

^{13}C NMR (100 MHz, $CDCl_3$) δ: 153.3, 140.1, 137.1, 136.6, 134.3, 133.2, 131.2 (t, $J_{C\text{-}F}$ = 8.2 Hz), 129.2, 129.1, 128.8, 128.6, 127.4, 120.7, 20.1.

MS (ESI) m/z: $[M+H]^+$ calcd for $C_{25}H_{20}N_2BF_2S_2$ 461.11, found 461.12; $[M+Na]^+$ calcd for $C_{25}H_{19}N_2BF_2S_2Na$ 483.09, found 483.10.

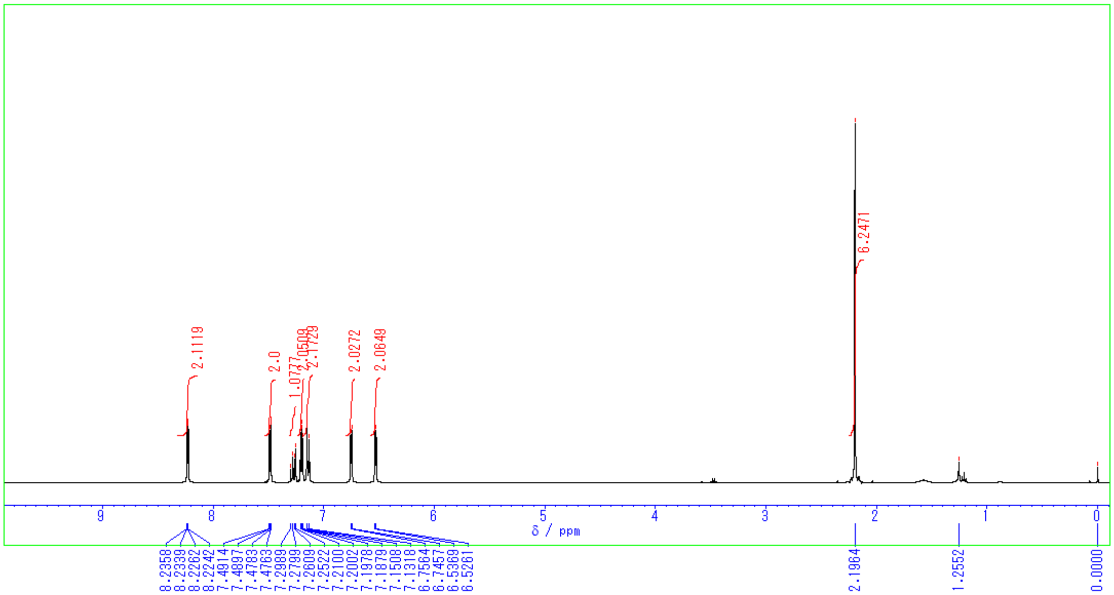

Figure S1. ^{1}H NMR spectrum of LD-TB (400 MHz, $CDCl_3$) at room temperature.

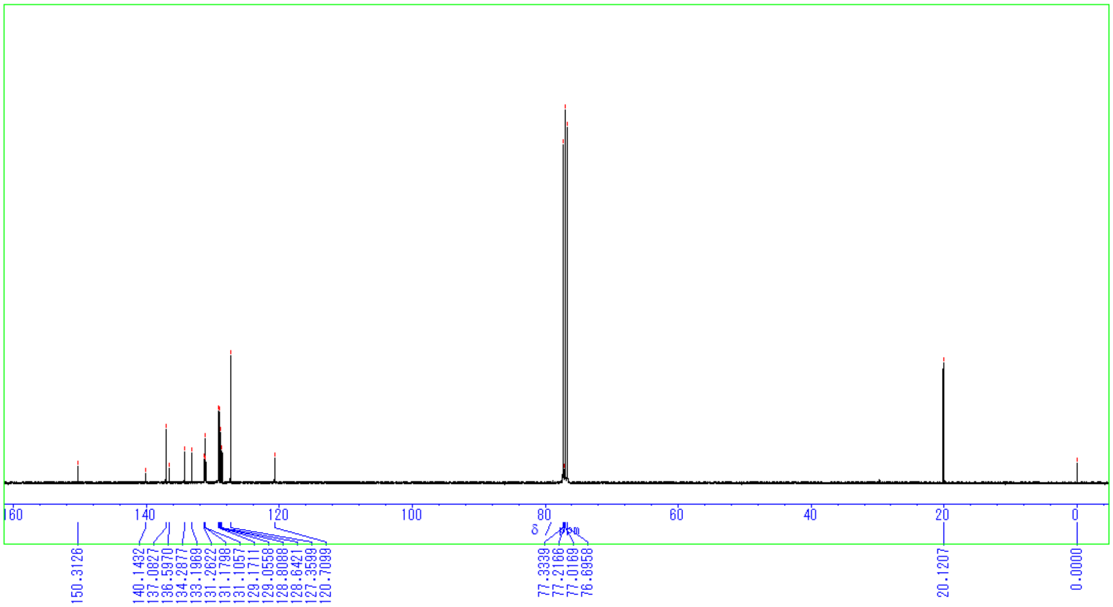

Figure S2. ^{13}C NMR spectrum of LD-TB (100 MHz, $CDCl_3$) at room temperature.

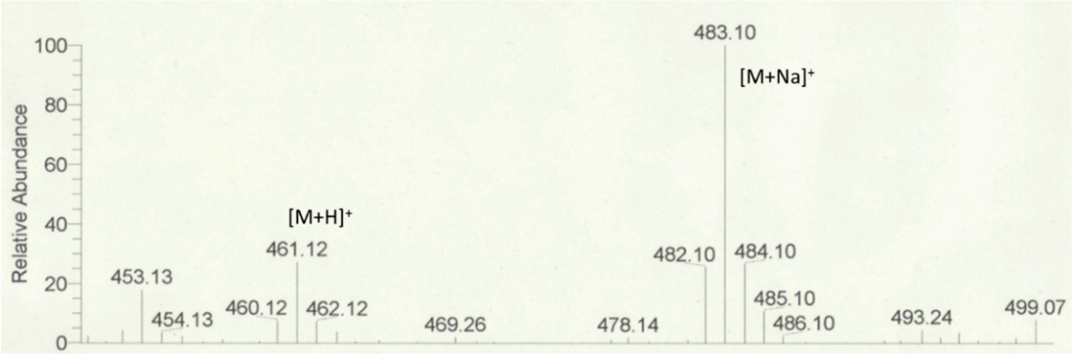

Figure S3. Mass spectrum of LD-TB.

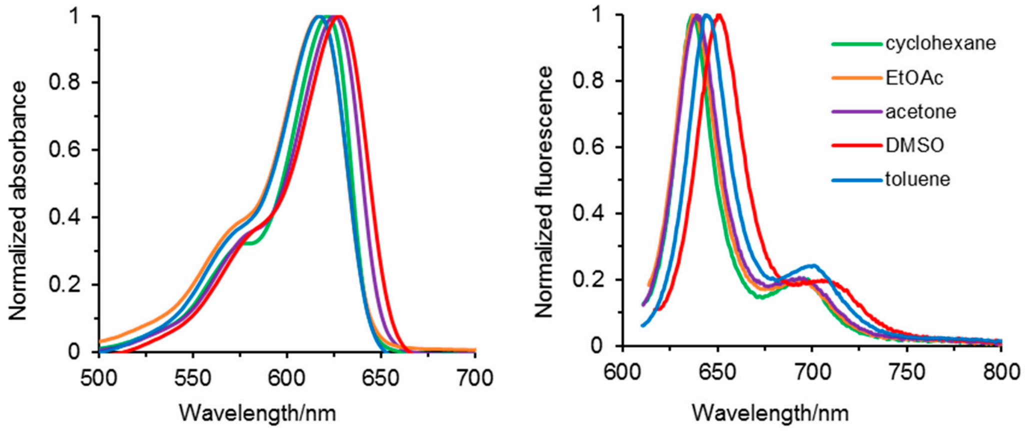

Figure S4. The normalized absorption spectra (left) and normalized emission spectra (right) of LD-TB (1 μM) in various organic solvents.

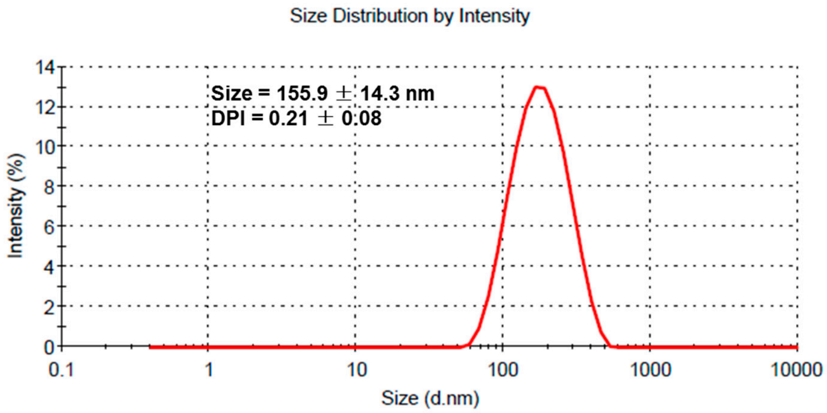

Figure S5. DLS measurements of LD-TB in 0.5% DMSO/H_2O at 0.05 μM.

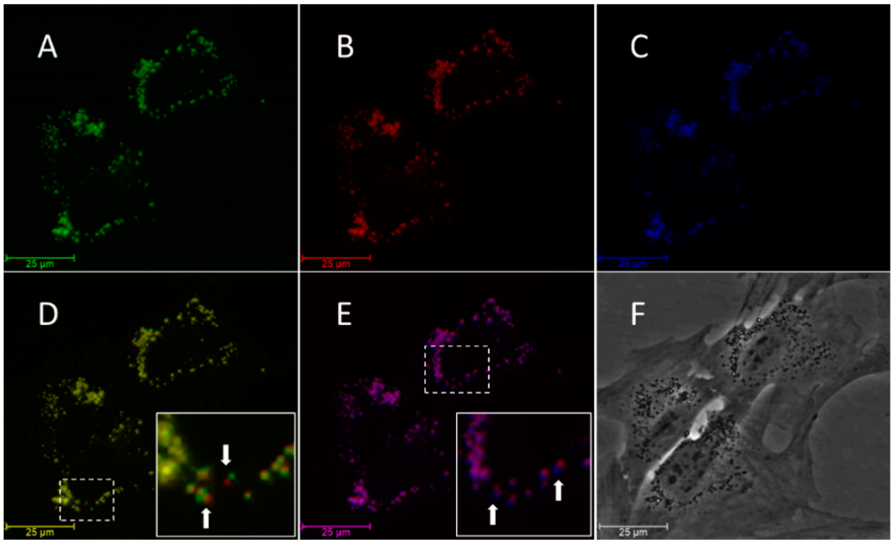

Figure S6. Fluorescence images of bovine cumulus cells stained with 0.05 µM LD-TB 30 min. Different false color (green, red, blue) are used to present the images captured at different times of 0, 1, 5 min (**A**–**C**). Merged images at different times, 0 and 1 min (**D**), 1 and 5 min (**E**), respectively. Bright field (**F**). Movement of the stained vesicles can be easily observed in the merged images. Scale bar 25 µm.

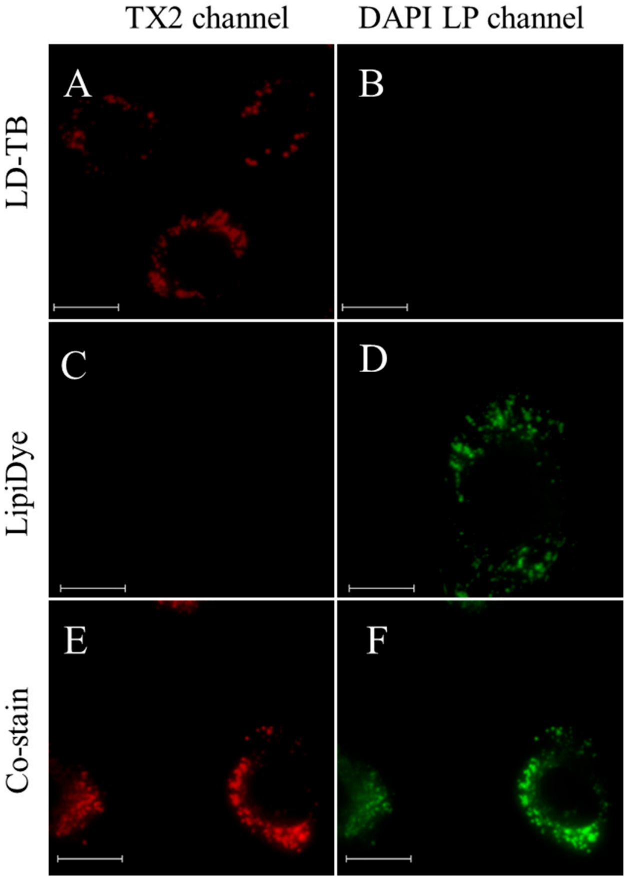

Figure S7. Fluorescence images of bovine cumulus cells: (**A** and **B**) stained with 0.05 µM LD-TB 30 min, (**C** and **D**) stained with 3 µM LipiDye 2 h, (**E** and **F**) stained with both dyes, firstly 3 µM LipiDye

1.5 h and then co-stain with 0.05 μM LD-TB for further 30 min. No cross-talk was observed in the colocalization experiments. Scale bar: 20 μm.

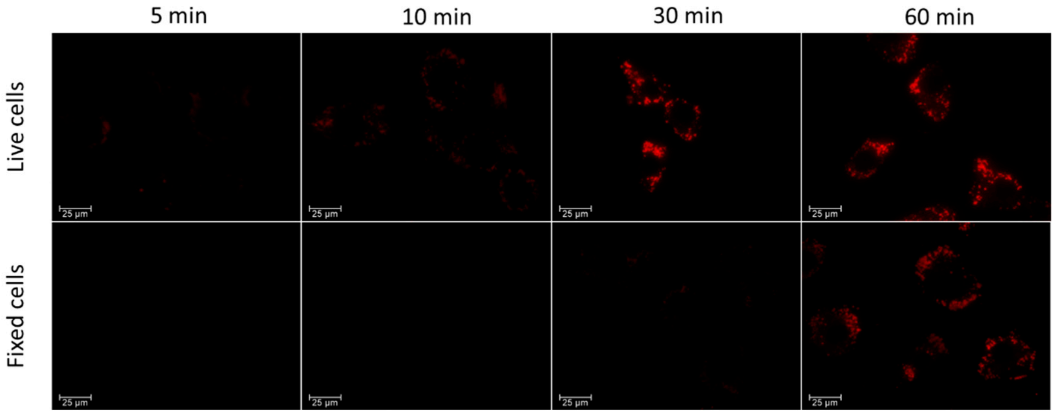

Figure S8. Images of live and fixed bovine cumulus cells stained with LD-TB (0.05 μM) for different period of time. For live cells, they were incubated at 38.5 °C. For fixed cell, they were fixed, washed and then incubated with LD-TB at room temperature. Conditions for images recording and post-processing were identical. Scale bar: 25 μm.

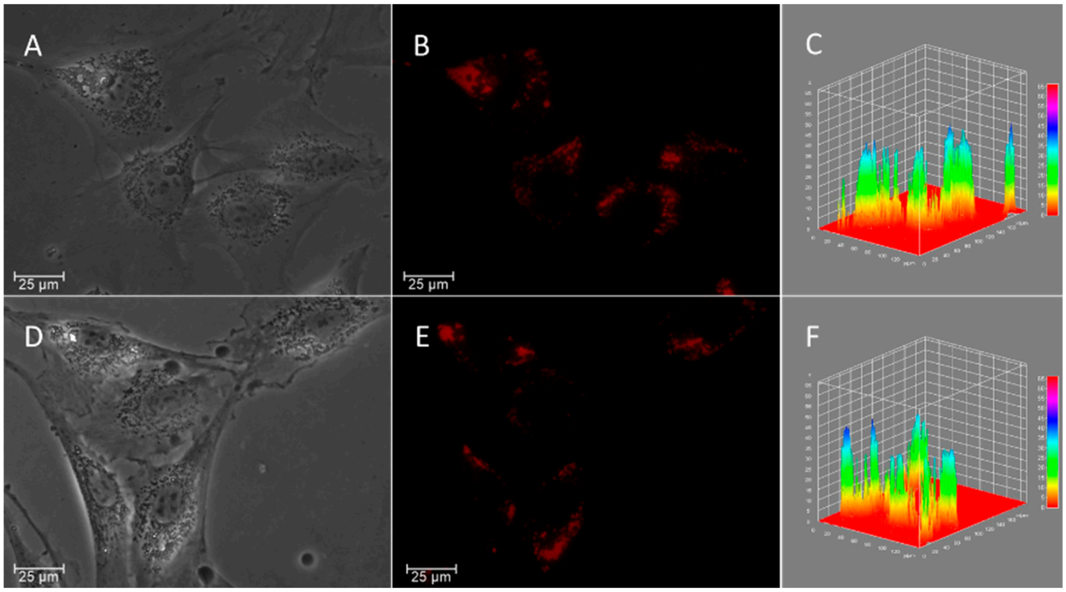

Figure S9. Images of bovine cumulus cells incubated with LD-TB (0.05 μM) for 30 min of live cells (**A**–**C**), and the images of cells incubated with LD-TB (0.05 μM) for 30 min of live cells and then fixed with PFA (**D**–**F**). (**A** and **D**) Bright field images. (**B** and **E**) Fluorescence images. (**C** and **F**) Image J 3D surface plot analysis. Conditions for images recording and post-processing were identical. No fluorescence quenching caused by fixation process was observed. Scale bar: 25 μm.

Reference

1. Li, G.; Otsuka, Y.; Matsumiya, T.; Suzuki, T.; Li, J.; Takahashi, M.; Yamada, K. A Straightforward Substitution Strategy to Tune BODIPY Dyes Spanning the Near-Infrared Region via Suzuki–Miyaura Cross-Coupling. *Materials* **2018**, *11*, 1297.

© 2020 by the authors. Submitted for possible open access publication under the terms and conditions of the Creative Commons Attribution (CC BY) license (http://creativecommons.org/licenses/by/4.0/).
